# An integrated systems-level model of ochratoxin A toxicity in the zebrafish (*Danio rerio*) embryo based on NMR metabolic profiling

**DOI:** 10.1038/s41598-022-09726-4

**Published:** 2022-04-15

**Authors:** Muhamed N. H. Eeza, Narmin Bashirova, Zain Zuberi, Jörg Matysik, John P. Berry, A. Alia

**Affiliations:** 1 Institute for Medical Physics and Biophysics, University of Leipzig, Leipzig, Germany; 2 Institute for Analytical Chemistry, University of Leipzig, Leipzig, Germany; 3 Leiden Institute of Chemistry, Leiden University, Leiden, The Netherlands; 4 Department of Chemistry and Biochemistry, Florida International University, Miami, FL USA; 5 Biomolecular Science Institute, Florida International University, Miami, FL USA

**Keywords:** Metabolomics, Diagnostic markers, Biochemical networks

## Abstract

Ochratoxin A (OTA) is one of the most widespread *mycotoxin* contaminants of agricultural crops. Despite being associated with a range of adverse health effects, a comprehensive systems-level mechanistic understanding of the toxicity of OTA remains elusive. In the present study, metabolic profiling by high-resolution magic angle spinning (HRMAS) NMR, coupled to intact zebrafish embryos, was employed to identify metabolic pathways in relation to a systems-level model of OTA toxicity. Embryotoxicity was observed at sub-micromolar exposure concentrations of OTA. Localization of OTA, based on intrinsic fluorescence, as well as a co-localization of increased reactive oxygen species production, was observed in the liver kidney, brain and intestine of embryos. Moreover, HRMAS NMR showed significant alteration of metabolites related to targeting of the liver (i.e., hepatotoxicity), and pathways associated with detoxification and oxidative stress, and mitochondrial energy metabolism. Based on metabolic profiles, and complementary assays, an integrated model of OTA toxicity is, thus, proposed. Our model suggests that OTA hepatotoxicity compromises detoxification and antioxidant pathways, leading to mitochondrial membrane dysfunction manifested by crosstalk between pathways of energy metabolism. Interestingly, our data additionally aligns with a possible role of mitochondrial fusion as a “passive mechanism” to rescue mitochondrial integrity during OTA toxicity.

## Introduction

The Food and Agriculture Organization estimated that approximately 25% of the world’s food crops are contaminated by one or more *mycotoxins* resulting from fungal contamination^1^. More recent surveys, however, suggest that this prevalence may be much higher with estimates ranging from 30 to 100% of foods and feeds contaminated at detectable levels^2–4^.

One of the most frequently reported mycotoxins found in agricultural products is ochratoxin, and specifically ochratoxin A (OTA) that is both the most common, and the most toxic, congener. Derived from species of *Aspergillus* and *Penicillium,* OTA is found as a contaminant of a wide range of food and feed crops including grains and fruits, as well as spices and other non-“food” crops (e.g., coffee), and a wide range of agricultural products derived from these (e.g., juices, wine, beer, cereals, bread). Concentrations of OTA in various grains, as well as products derived from these (e.g., flour, bread, cereals), have been typically reported, for example, in the parts-per-billion (ppb, i.e., µg/kg) range, however, have been reported at levels more than 2 mg/kg^5^. Regulatory limits for OTA in agricultural products are, in fact, generally in the < 10 µg/kg range. In addition to plant crops, animal products including milk, eggs and meat may accumulate mycotoxins (via contamination of animal feeds) with levels detected, likewise, in the ppb (i.e., µg/kg) range^5^. And alongside consequent potential for human exposure to OTA, considerable economic losses in the livestock industry are, therefore, associated with OTA contamination including decreased feed intake, and consequent reductions in growth, and increased mortality rates, of animals, as well as medical expenses of animals, and mitigation costs^6,7^.

Toxicity of OTA, and associated pathologies including carcinogenicity, are primarily associated with liver and kidney as target organs, however, a wide range of toxicities including immunotoxicity, teratogenicity and neurotoxicity have been reported. Possible mechanisms of action demonstrated for OTA have included genotoxicity, specifically by way of DNA adducts, and consequent single-strand breaks^8,9^, inhibition of protein synthesis^10^, and impairment of mitochondria with consequent impacts on cellular bio-energetics, and associated production of reactive oxygen species (ROS), and subsequent oxidative stress^11,12^. Disruption of transcription regulation via *nuclear factor erythroid 2-related factor 2* (Nrf2) has been identified, in several studies, as a molecular target of OTA^13,14^. Effects on Nrf2-based signal transduction pathways are likely to be broadly important as it is a key transcription factor for regulating antioxidant, phase-II detoxification (i.e., conjugation and removal) and other cytoprotective pathways^15^. Numerous studies have, in addition, suggested a role of phase-I detoxification pathways including both potential upregulation of relevant enzymes, and particularly, cytochrome P450 (CYP), and identification of numerous biotransformed metabolites of OTA^16^. While much has been gleaned from these previous studies, a *systems-level* understanding of OTA toxicity which incorporates these diverse, interrelated cellular, molecular and biochemical targets has, however, remained elusive.

Early life stages (i.e., embryos and larvae) of the zebrafish (*Danio rerio*) have become widely established as a toxicological model for a range of environmental toxicants including mycotoxins and, indeed, OTA^17–21^. Several previous studies have characterized OTA toxicity, typically in the sub-micromolar range, in embryo and larval stages of the zebrafish including acute lethality, developmental dysfunction and neurobehavioral effects, as well as toxin-specific targets including hepatoxicity and nephrotoxicity^17–20,22–24^. The capacity of this model system is potentiated not only by the availability of a wide range of scorable toxicological endpoints, but also a growing range of biochemical and molecular technologies including “omics” approaches which have been adapted to zebrafish embryos and larvae^25^. Among these, are several mass spectrometric (MS) and nuclear magnetic resonance (NMR) spectroscopic tools for metabolomics. And of these, *high-resolution magic angle spinning* (HRMAS NMR) for metabolic profiling has been recently demonstrated as a powerful technique for assessment of toxicants in the zebrafish embryo model from a *systems biology* perspective^21,26–28^. In the present study, we employed an approach based on HRMAS NMR metabolic profiling, coupled to zebrafish embryonic stages, as well as other complementary techniques, to identify and characterize relevant metabolic pathways, and subsequently, develop an integrated systems-level model, of OTA toxicity.

## Results and discussion

### Toxicity of OTA in the zebrafish embryo model

**Figure 1:**
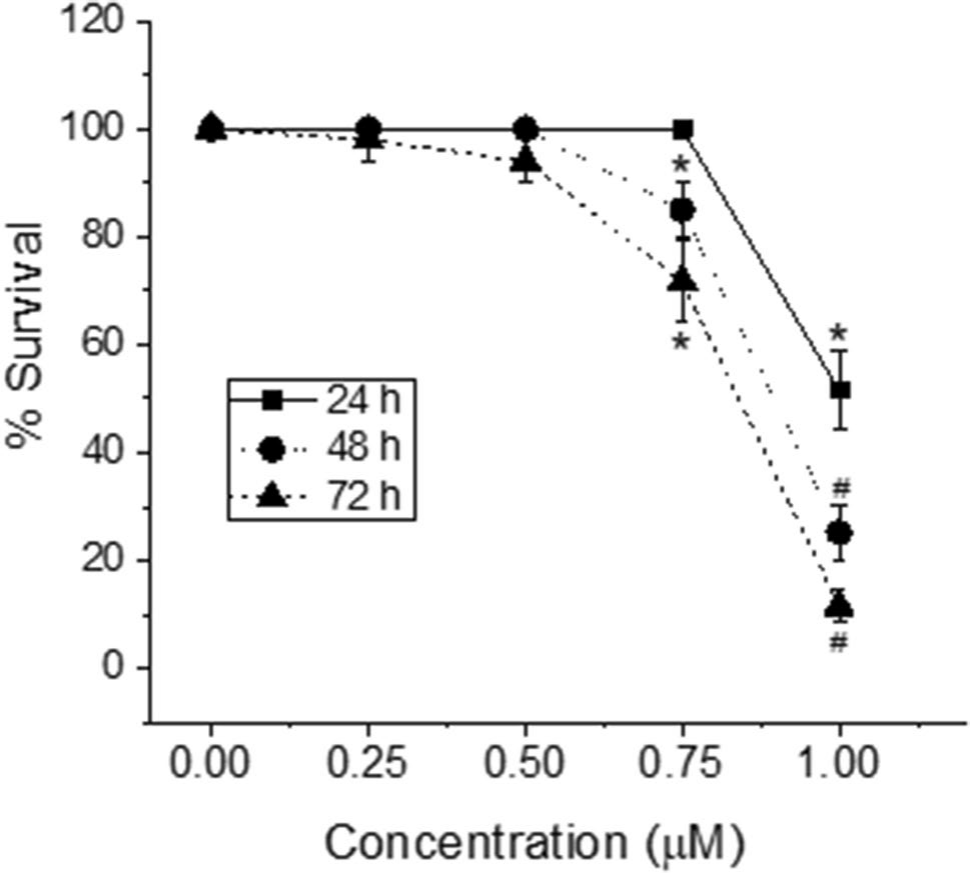
Concentration- and time-dependent toxicity of OTA in the zebrafish embryo model. Zebrafish embryos (72 hpf) were exposed to different concentrations of OTA (0, 0.25, 0.5, 0.75 and 1.0 µM). Percentage survival of the embryos were recorded after 24 h, 48 h and 72 h exposures. Concentration–dependent toxicity was observed, and increased with exposure time. Values shown are the mean ± standard deviation (n = 6 replicates per group, and 10 embryos per replicate). *P < 0.05 and # *P* < 0.01 as compared to untreated controls.

**Figure 2:**
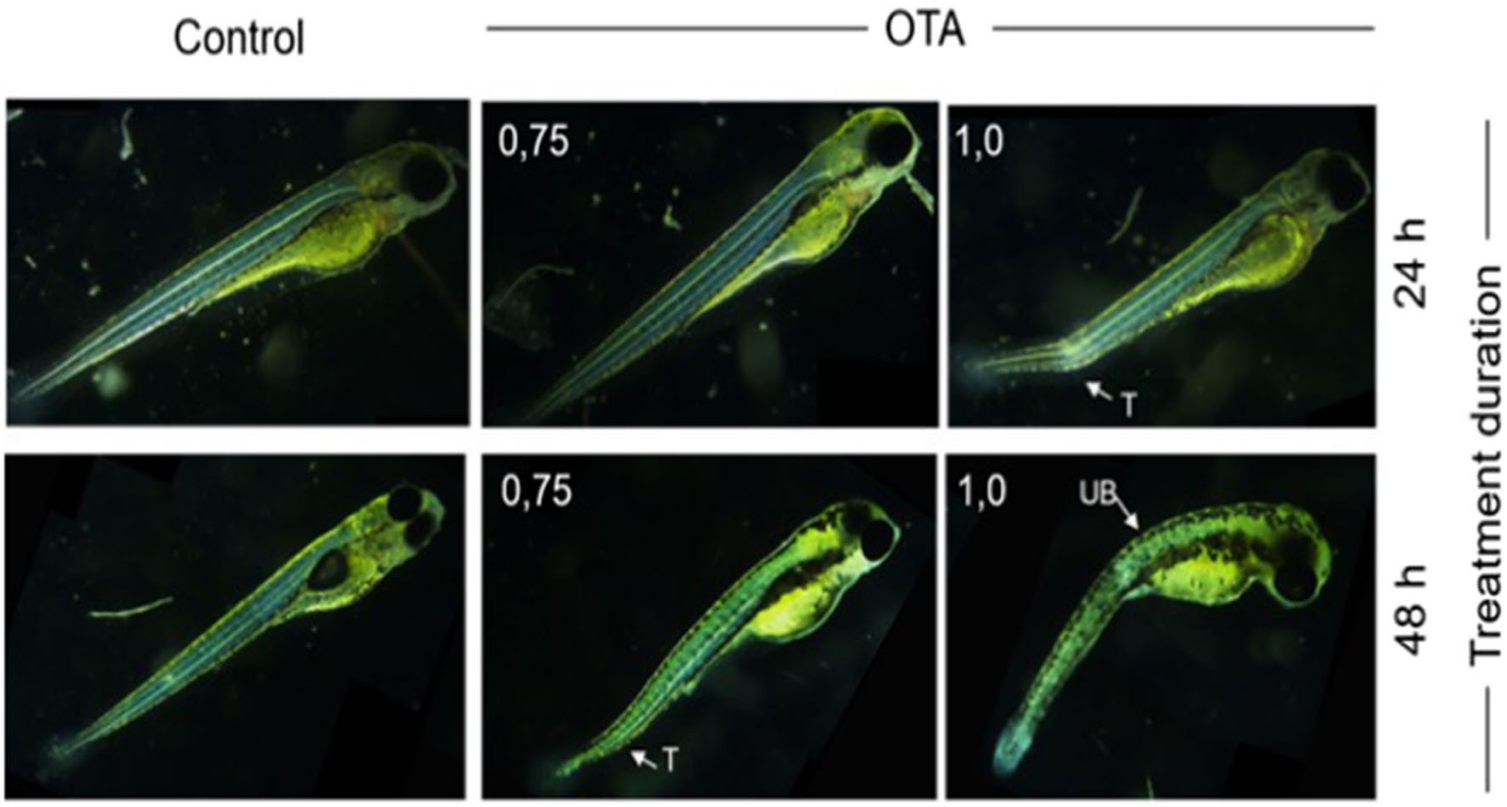
Representative images showing developmental deformities of zebrafish embryos. The embryos were exposed (at 72 hpf) to 0.75 µM or 1.0 µM OTA for a duration of 24 h or 48 h, compared to untreated (i.e., Control) embryos. Images were taken at 96 hpf (i.e., 24 h, upper) and 120 hpf (i.e., 48 h, lower). Deformities include bending of upper body (UB) and tail (T). Scale bar = 500 µm.

Dose- and time-dependent embryotoxicity including lethality, and developmental impairment, was observed for zebrafish embryos exposed at 72 h post-fertilization (hpf) to OTA (Figs. 1 and 2). Significant mortality (*P* < 0.05), compared to untreated controls, was observed at 1.0 µM within 24 h of exposure, and the lowest-observable-adverse-effect levels (LOAEL) decreased to 0.75 µM and 0.5 µM after 48 h and 72 h of exposure, respectively (Fig. 1). Developmental deformity specifically characterized by malformation of the head, and curvature of the tail and upper body (Fig. 2), was observed for 20–30% of surviving embryos at, or above, 1.0 µM and 0.75 µM OTA, respectively, for 24 h and 48 h exposures. Embryotoxicity observed in this study quantitatively aligns with previous reports of OTA toxicity in the zebrafish embryo model^17,19^. Haq et al.^16^, for example, reported developmental deformities for continuously exposed embryos with LOAEL of 1 µM and 0.1 µM OTA at 24 and 96 h, respectively, as well as median lethal concentrations (LC_50_) of 0.25 µM at 96 hpf.

Based on these results, an exposure time of 72–96 hpf, and exposure concentration of 1.0 µM, was selected for subsequent metabolic profiling studies (as discussed below). This exposure time and concentration approximates the median lethality in zebrafish embryos. Moreover, accompanying the observed toxicity, relevant organ systems including CNS, gastrointestinal system and liver (as previously reported targets of OTA^18,20,24,29,30^) are generally differentiated at this developmental stage, and loss of the chorion as a potential barrier to OTA uptake following hatching generally occurs, likewise, at approximately 72 hpf. Exposure of embryos at this development stage in subsequent aspects of the study, therefore, enabled both improved uptake potential (i.e., post-hatch loss of chorion), and assessment of the possible targeting of these fully differentiated organ systems.

### In vivo localization of OTA in exposed zebrafish embryos

**Figure 3:**
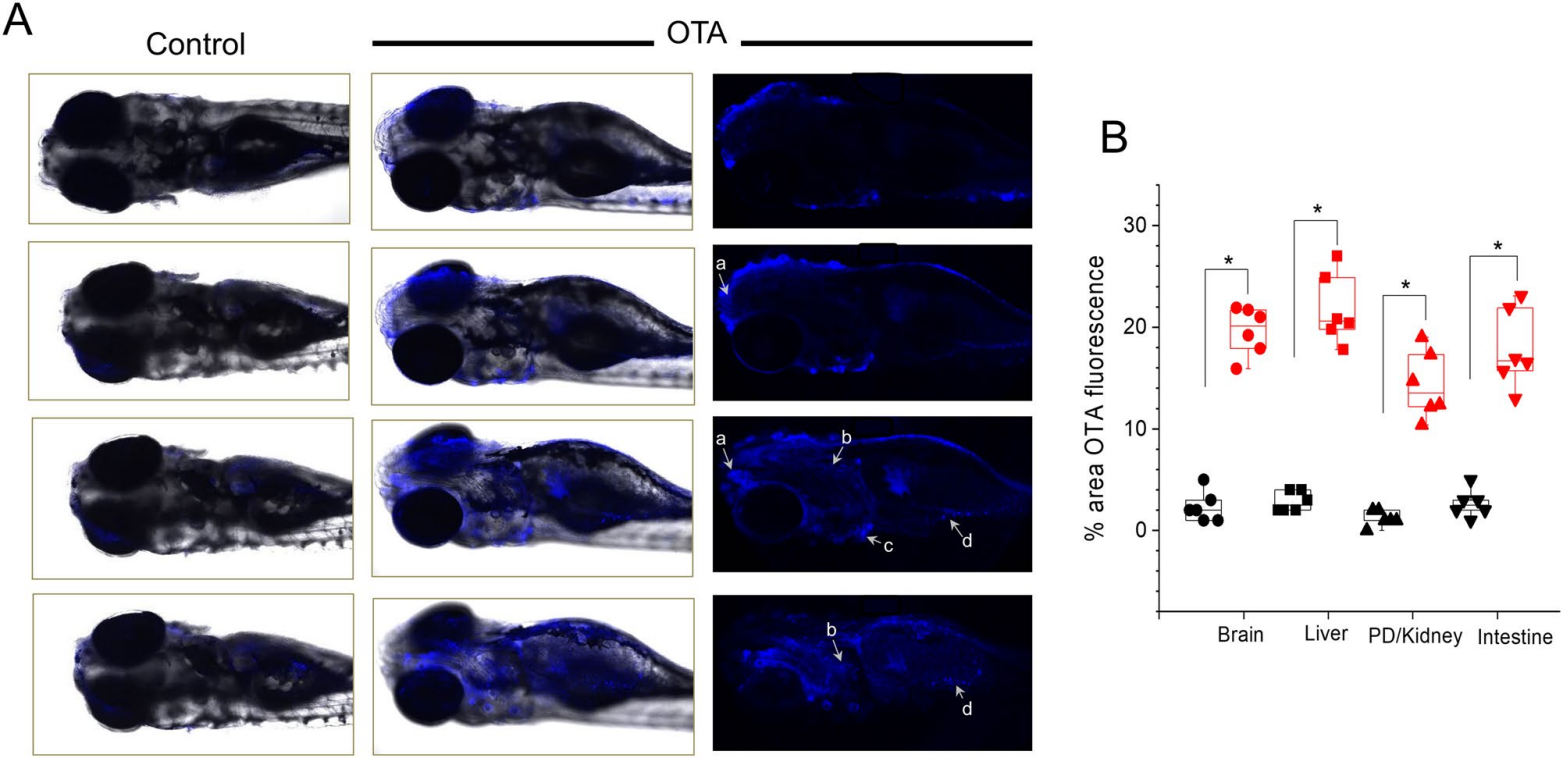
Distribution of ochratoxin A (OTA) visualized based on its intrinsic fluorescence. (**A**) The distribution of OTA is shown in the body of 120-hpf zebrafish embryos exposed to 4 µM OTA for 48 h, in comparison to untreated (i.e., “Control”) 120-hpf embryos. Left and middle columns show successive slices through the Control and OTA-treated embryos, respectively, with fluorescence (blue) overlayed on bright field images. Right column shows corresponding fluorescent images of ochratoxin-treated embryos without overlay. As indicated, OTA is accumulated in brain (a), pronephric duct/kidney (b), liver (c) and intestine (d). Images were acquired using inverted laser-scanning confocal microscope (Leica DMi8/TL LED, Leica Microsystems CMS GmbH) with an excitation wavelength of 380 nm, and emission wavelength of 460 nm. A Leica HC PL Apo CS2 (10x/0.15 Dry) objective, and Leica Application Suite X (LAS X) software package version 3.1.5, were used to capture images. (**B**) Quantitative analysis of OTA fluorescence in brain, liver, pronephric duct (PD)/kidney and intestine of OTA treated zebrafish embryos (red) as compared to untreated control (black) (n = 6 per group). *P < 0.05.

Owing to the conjugated isocoumarin moiety of the molecule, OTA is intrinsically fluorescent in the long-wave UV range. Indeed, this fluorescence has been widely coupled to chromatographic separation as a highly sensitive means to detect and quantify OTA^31^. Enabled by the near transparency of the zebrafish embryo, it was found that OTA at suitably high concentrations (≥ 4 µM) could be visualized by confocal microscopy in intact embryos (Fig. 3). This novel means of in vivo observation of the distribution of OTA within embryos is previously unreported in the literature. And in the current study, enabled assessment of toxicologically relevant endpoints (e.g., ROS production) in relation to the observed patterns of distribution.

Accordingly, in vivo visualization was able to identify primary localization of OTA to liver and pronephric duct/kidney, as well as intestine and brain regions, of 72 hpf embryos (Fig. 3). Localization to these organ systems is consistent with numerous studies which have, likewise, reported targeting of OTA to liver, kidney and intestines in other animal models including both presence and toxicity in these systems^32–34^, as well as several recent studies which have reported both distribution of OTA in multiple regions of the brain, and corresponding neurobehavioral effects^35–37^. The observed pattern of distribution is, furthermore, conspicuously aligned with an established role of organic anion-transporting polypeptides (OATP, i.e., *SLCO* superfamily) and organic anion transporters (OAT, i.e., *SLC22A* superfamily) in the cellular uptake of OTA^38–40^, which have been shown to be primarily localized to liver, kidney, intestine and brain in the zebrafish^41,42^. The observed distribution of OTA to these organ systems supports alteration of metabolic profiles, and other toxicological endpoints (e.g., oxidative stress), subsequently observed in the current study.

### Alteration of metabolic profiles by OTA

**Figure 4:**
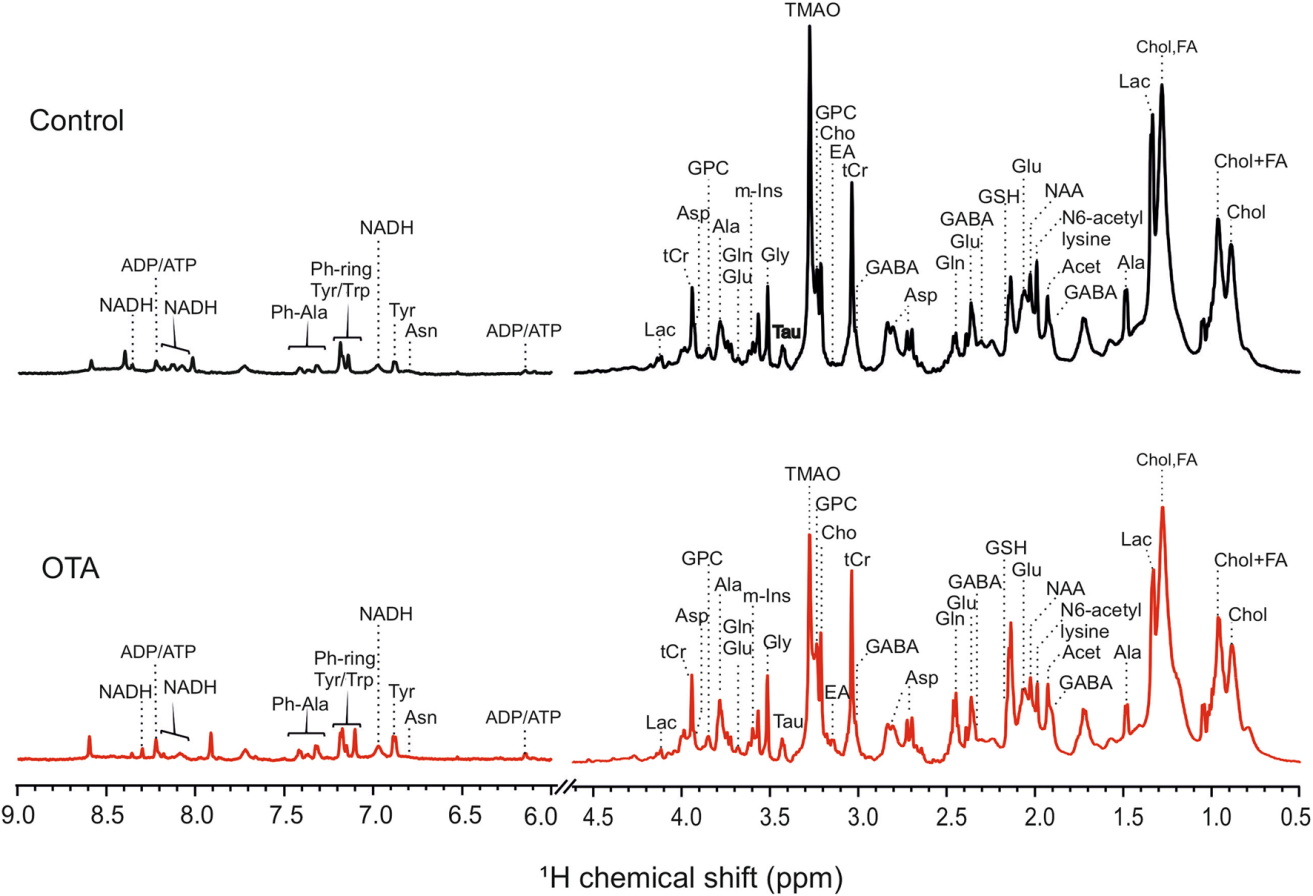
High-resolution magic angle spin (HRMAS) NMR based metabolic profiling. Representative HRMAS NMR spectra of (**A**) Control and (**B**) OTA (1 µM) exposed zebrafish embryos (72 hpf) treated for 24 h.

**Figure 5:**
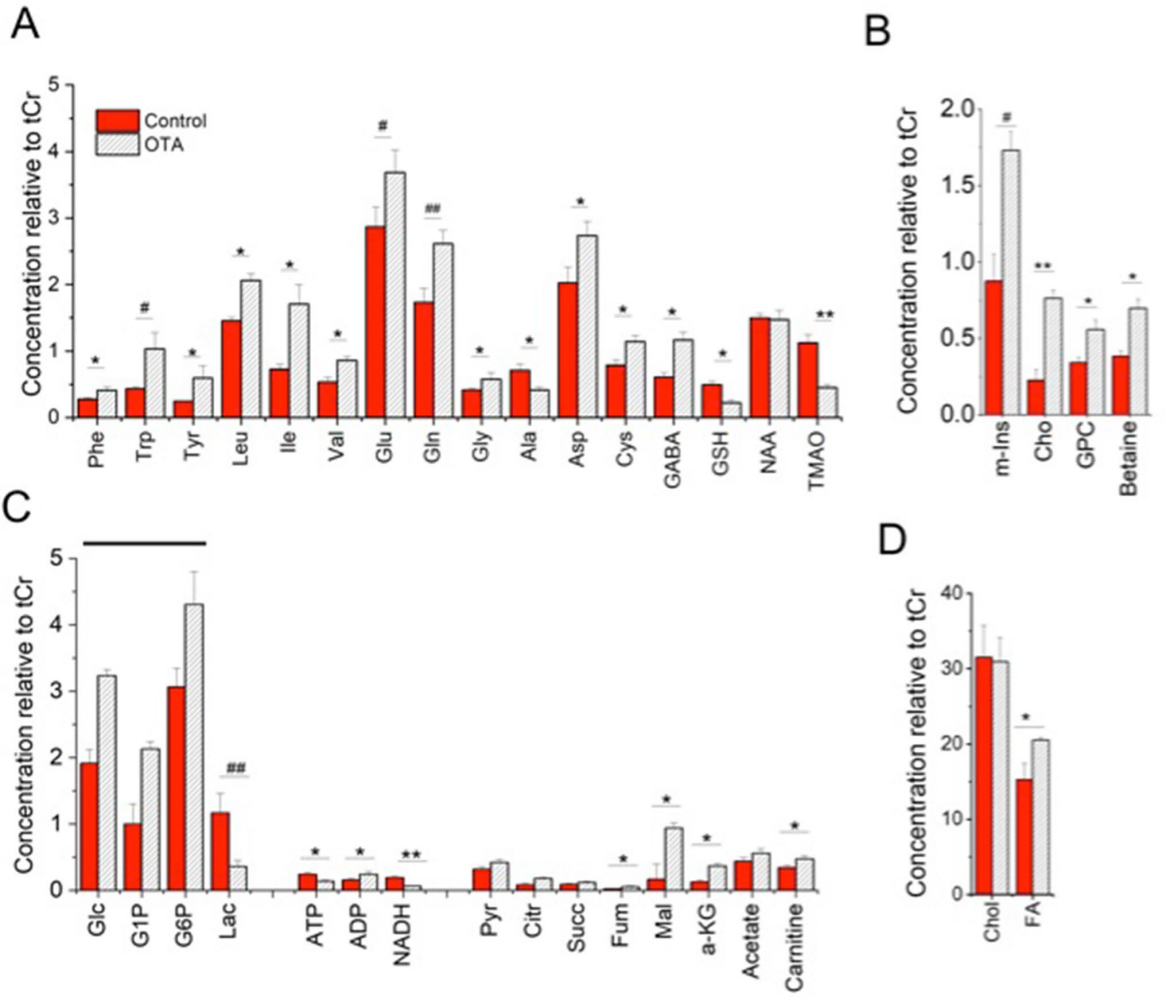
Effect of OTA treatment on the metabolic profile of intact zebrafish embryos. Zebrafish embryos (72 hpf) were exposed to 1 µM OTA or solvent vehicle (“Control”), for 24 h. Concentrations of metabolites relative to total creatine (tCr) are shown, and include (**A**) amino acids and related metabolites (**B**) polar head-groups of membrane phospholipids; (**C**) metabolites associated with energy metabolism; and (**D**) lipids, i.e., fatty acids and cholesterol. For statistical analysis, one-way ANOVA with a Tukey post-hoc correction for multiple comparisons were performed using OriginPro v. 8 (Northampton, MA, USA). Values shown are the mean ± standard deviation (n = 6). ## *P* < 0.0001, # *P* < 0.001, ** *P* < 0.01 and *P < 0.05. *Note* As indicated by line, Glc, G1P and G6P could not be unambiguously identified by HRMAS NMR, so statistical significance of differences is not given, however, all three were resolved, and relative changes (i.e., increases) were confirmed, by 2D COSY experiments (see Supplementary Fig. S1). Abbreviations: Phe = phenylalanine; Trp = tryptophan; Tyr = tyrosine; Leu = leucine, Ile = isoleucine; Val = valine; Glu = glutamate; Gln = glutamine; Gly = glycine; Ala = alanine; Asp = aspartate; Cys = cysteine; GABA = γ-aminobutyric acid; GSH = glutathione; TMAO = trimethylamine *N*-oxide; Glc = glucose; G1P = glucose-1-phosphate; G6P = glucose-6-phosphate; Lac = lactate; ATP = adenosine triphosphate; ADP = adenosine diphosphate; NADH/NAD +  = reduced/oxidized nicotinamide adenine dinucleotide; m-Ins = myo-inositol; Cho = choline; GPC = glycerophosphocholine; Pyr = pyruvate; Cit = citrate; Suc = succinate; Fum = fumarate; Mal = malate; a-KG = alpha ketoglutarate; Chol = cholesterol; FA = fatty acids.

Toward elucidation of toxicologically relevant pathways, a previously developed^26,27^ method of HRMAS NMR for metabolic profiling of intact zebrafish embryos was employed to identify metabolites altered by exposure to OTA. Metabolic profiles of intact embryos (72 hpf) treated with 1 µM OTA for 24 h, and assessed by 1D ^1^H HRMAS NMR, enabled effective resolution of metabolic profiles for both OTA-treated and control embryos (Fig. 4). Multivariate analysis of the HR-MAS NMR spectra using partial least square-discriminant analysis (PLS-DA) modelling provided statistical discrimination of spectral regions, and corresponding compounds, mainly responsible for distinction (Fig. S1) of the quantitative differences in metabolites between OTA-exposed and control embryos. The quantitative analysis of metabolites between control and OTA exposed embryos shows significant alteration of 30 metabolites (Fig. 5).

Numerous metabolites associated with interrelated pathways of energy metabolism including corresponding pathways of carbohydrate, amino acid and lipid catabolism were altered in OTA-exposed embryos. Both ATP and NADH, as the primary currency of cellular energetics, were significantly decreased in OTA-treated embryos. Concomitantly, significant increases in several amino acids including tryptophan (Trp), tyrosine (Tyr), leucine (Leu), isoleucine (Ile), valine (Val), glutamate (Glu), glutamine (Gln), glycine (Gly), cysteine (Cys), aspartate (Asp) and phenylalanine (Phe) were observed, consistent with impairment of amino acid catabolism, whereas a significant *decrease* was, in contrast, notably measured for alanine (Ala). Interestingly, the non-proteinogenic amino acid, γ-aminobutyric acid (GABA), also significantly increased (*P* < 0.05); although, GABA is most often associated with its function as neurotransmitter, emerging evidence suggests a possible role in hepatic damage^43^. Metabolites associated with carbohydrate metabolism were also altered by OTA treatment including a significant decrease in lactate (Lac), and seemingly increased levels of glucose (Glc) and associated metabolites, specifically including glucose-6-phosphate (G6P) and glucose-1-phosphate (G1P). Given that Glc and these phosphorylated metabolites were challenging to unambiguously resolve in HRMAS NMR spectra, the identity and relative increase (between treated and controls embryos) of all three were additionally confirmed with measurement of two-dimensional homonuclear correlation spectroscopy (^1^H-^1^H COSY) as described previously^27^ (Supplementary Fig. S1). As shown in Fig. 5, a significant increase in fatty acids (FA), but no significant difference in cholesterol (Chol), was also observed; and notably, alongside increased FA, a significant increase in carnitine as recognized co-factor in the β-oxidation of fatty acids (but not cholesterol catabolism) was measured.

**Figure 6:**
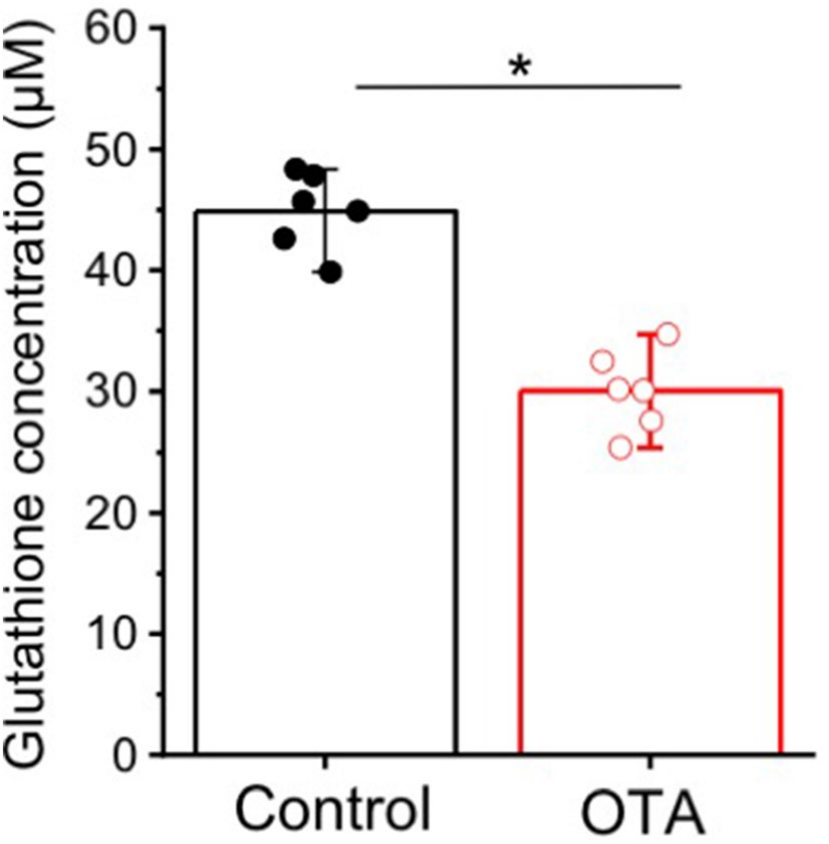
Glutathione levels in extracts of zebrafish embryos (72 hpf) exposed to OTA (1 µM for 24 h) as compared to Control embryos. Glutathione (GSH) levels were analysed by using GSH assay kit from Sigma-Aldrich. Significant reduction of GSH (*P < 0.001; n = 6) in OTA-treated embryo is clearly observed.

**Figure 7:**
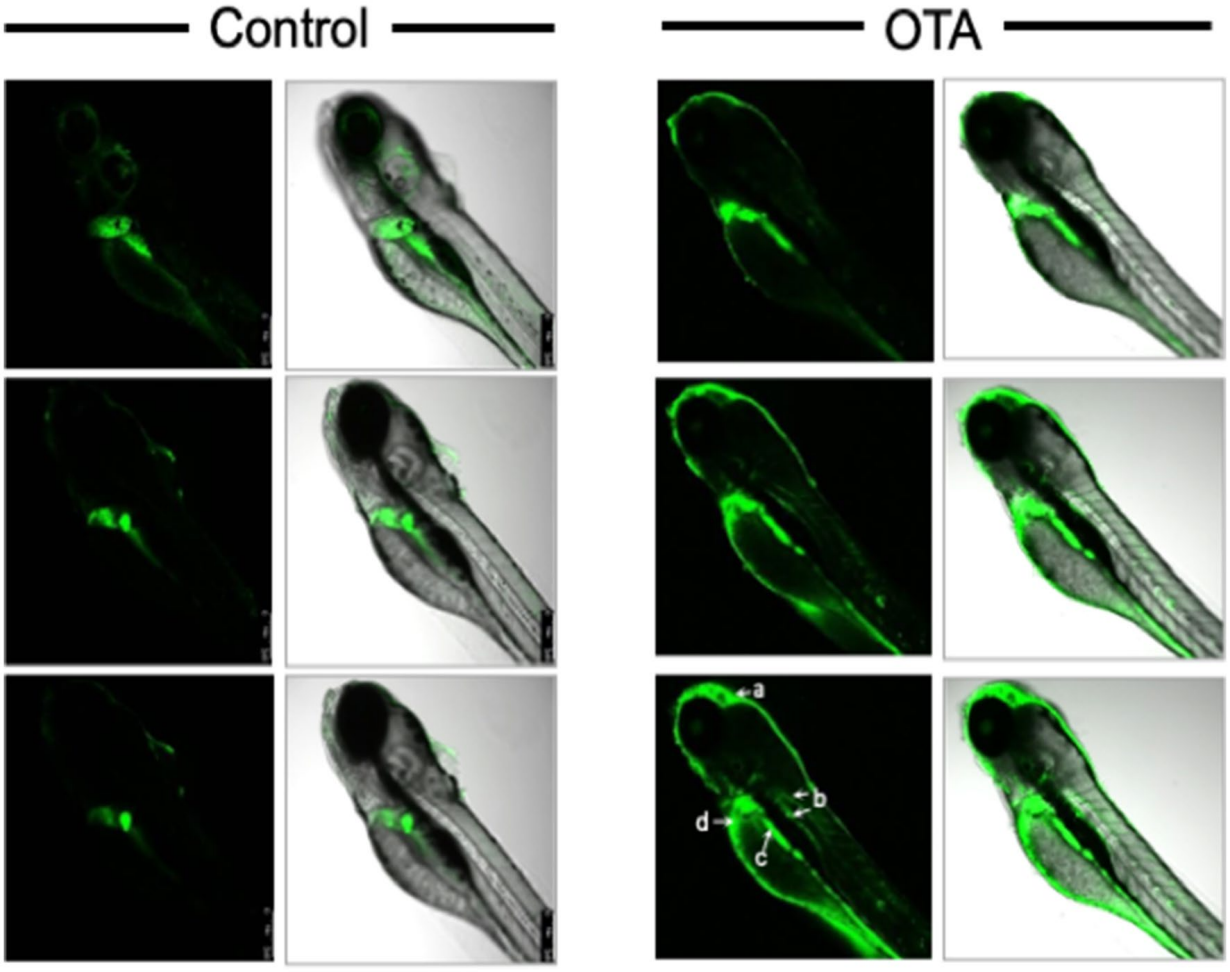
Localization of reactive oxygen species (ROS) production in zebrafish embryos exposed to OTA. ROS production was localized in zebrafish embryos (72 hpf) exposed to OTA (1 µM for 24 h) as compared to control embryos. Successive slices are shown through embryos (at 96 hpf) incubated for 60 min in CM-H2DCFA (10 μM) in rearing medium. Green fluorescence images (columns 1 and 3), and overlay with bright-field image (columns 2 and 4), are shown. As can be seen, increased ROS was clearly observed in brain (a), pronephric duct/kidney (b), and intestine (c), as well as liver.

A second general trend was alteration of metabolites directly or indirectly associated with oxidative stress and detoxification pathways. A significant decrease in glutathione (GSH) as both an antioxidant, and key metabolite in phase-II detoxification, was measured by HRMAS NMR following OTA exposure (compared to controls). Decreased GSH has been consistently measured alongside increased levels of ROS, for OTA in a range of toxicological systems^44^. The decrease in GSH was further confirmed, in the present study, by ex vivo colorimetric assay (Fig. 6), and moreover, increased ROS production was observed in vivo (Fig. 7), and specifically localized to organ systems coincident with distribution of OTA (i.e., liver, kidney, intestine and brain) in exposed embryos (Fig. 3). Consistent, albeit indirectly, with a role of oxidative stress (i.e., ROS), and antioxidant and detoxification pathways, significant increases were observed for glycerophosphocholine (GPC), choline (Cho) and myo-inositol (m-Ins) which are typically associated with polar headgroups of phospholipids, and betaine as a biosynthetic product of choline, whereas trimethylamine *N*-oxide (TMAO) was significantly decreased. Increased levels of polar headgroup molecules have been suggested in previous studies^45^ to be an indicator of disruption of cell membranes, and specifically hydrolysis of phospholipids, which can occur as a consequence of oxidative stress (e.g., lipid peroxidation). Interestingly, betaine (although associated with a range of cellular functions) has been recently proposed to serve a role in the regulation of mitochondrial function including, in particular, fusion/fission^46^ which, in turn, has been linked to oxidative stress^47^. On the other hand, TMAO has been proposed as potential biomarker of the impairment of liver (discussed below) where pathways of phase-I and II detoxification of xenobiotics are generally localized, and specifically, where phase-I oxidation to produce TMAO exclusively occurs.

**Figure 8:**
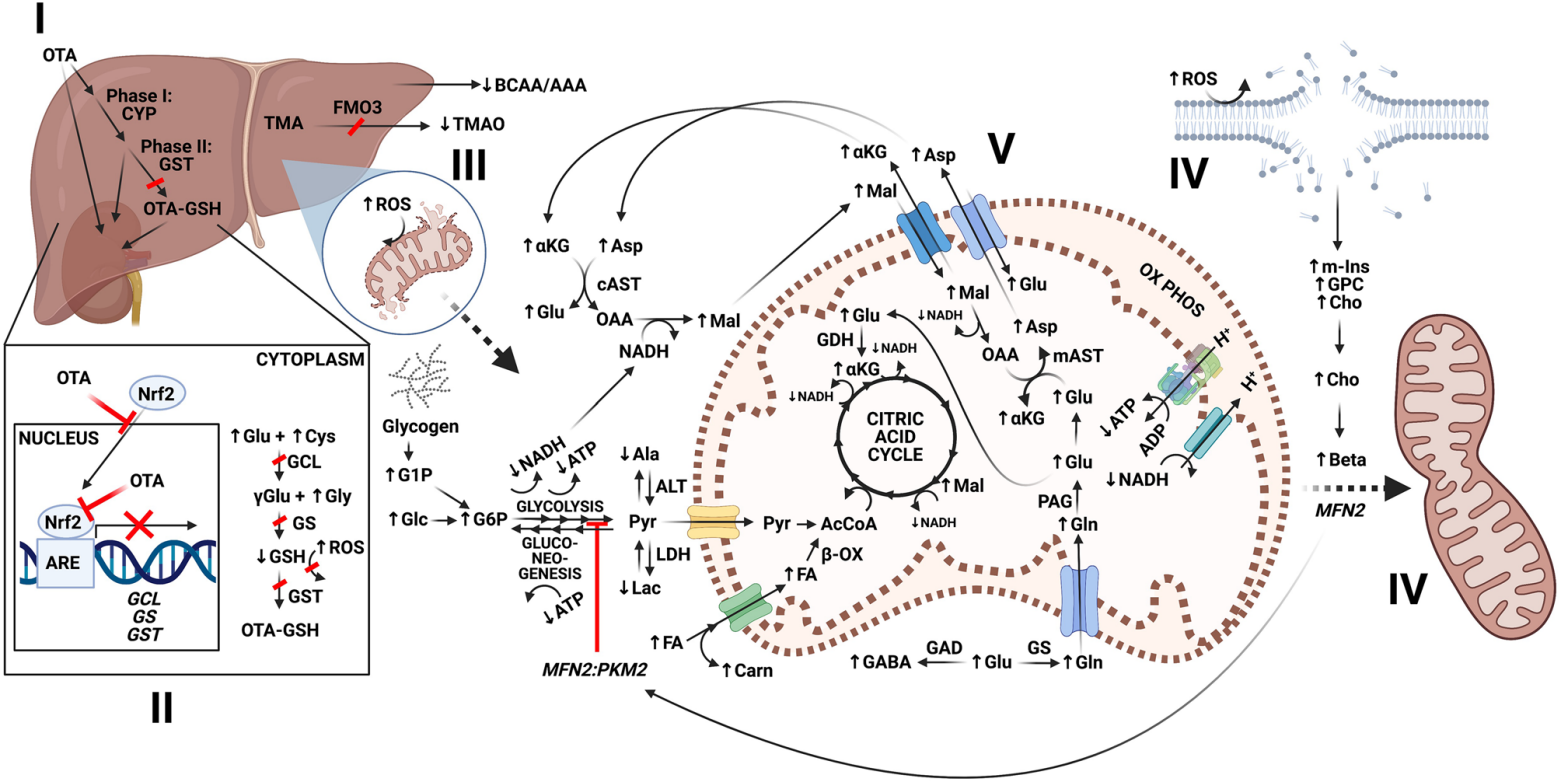
An integrated model of the hepatotoxicity mechanism of ochratoxin A (OTA) in relation to observed changes in metabolic alterations. In liver, OTA affects detoxification pathways (**I**) associated with disruption of Nrf2 that is a key transcriptional factor for regulating detoxification pathways, and the synthesis of GSH as an antioxidant (**II**). This is evident by a decrease in GSH, and increase in its precursors Cys, Glu and Gly. The depletion of GSH leads to impaired redox homeostasis, and an increased production of ROS, and consequently, lipid peroxidation and membrane damage especially affecting mitochondrial membrane integrity (**III**). Mitochondrial membrane hydrolysis is reflected by an increase in Cho, GPC, and m-Ins, as polar headgroups of membrane phospholipids (**IV**). The loss of membrane integrity leads to impairment of the mitochondrial membrane potential (MMP), with consequent reduction of oxidative phosphorylation as reflected by decrease in ATP. Several pathways upstream of oxidative phosphorylation are consequently affected, as seen by changes in metabolites associated with glycolysis and/or gluconeogenesis, the malate/aspartate shuttle, β-oxidation of fatty acids, glutaminolysis, and the citric acid cycle (**V**). The energy deficiency and hindered oxidative phosphorylation activates mitochondrial fusions, possibly via elevated levels of betaine, as a “passive mechanism” to rescue mitochondrial integrity and energy metabolism (**VI**). Observed increases and decreases in metabolites shown by arrows (i.e., ↑ and ↓, respectively). Abbreviations: CYP = cytochrome P450; GST = glutathione-S-transferase; TCA = Tricarboxylic acid cycle, i.e., citric acid cycle; AcCoA = acetyl CoA; OxPhos = oxidative phosphorylation; PL = phospholipids; BCAA/AAA = ratio of branched chain and aromatic amino acids; FMO3 = flavin-containing monooxygenase 3; LDH = Lactate dehydrogenase; ALT = alanine aminotransferase; TMA = trimethylamine; ARE = antioxidant response elements; OAA = oxaloacetate; cAST = cytosolic Aspartate transaminase; mAST = mitochondrial Aspartate transaminase; MFN2 = mitofusin-2; PKM2 = pyruvate kinase; PAG = phosphate-activated glutaminase; β-ox = Beta oxidation; GAD = glutamate decarboxylase; GS = glutamine synthetase; for other metabolite abbreviations, see legend for Fig. 5.

Alterations in metabolites are, therefore, consistent with multiple, recognized targets of OTA toxicity including (1) organ-specific targeting of liver and associated systems, e.g., kidney and intestine; (2) corresponding effects on antioxidant and detoxification pathways; and (3) specific targeting of mitochondria in relation to cellular bioenergetics. Taken together, an integrated systems-level model of OTA toxicity is proposed (Fig. 8).

### Liver as a target of OTA in zebrafish embryos

Although in vivo visualization studies observed apparent OTA fluorescence in liver, kidney, intestine and brain (Fig. 3), multiple metabolic alterations observed in the present study are particularly aligned with targeting of liver, and the established hepatotoxicity of OTA^48^. Similarly consistent with this, disruption of liver development by OTA was previously identified in the zebrafish embryo^20^. Of the altered metabolites, decreased TMAO is perhaps most directly indicative of hepatotoxicity: while its precursor, trimethylamine (TMA), is produced by gut microbiota (from various substrates, e.g., choline, carnitine, lecithin), subsequent *N*-oxidation (to TMAO) by flavin-containing monooxygenase 3 (FMO3) occurs exclusively in the liver^49^ (Fig. 8). And accordingly, TMAO has been suggested in previous studies^50^ including similar, recent metabolomics studies of other hepatotoxins in zebrafish^28^ as a biomarker of hepatic function.

Targeting of the liver and hepatotoxicity is, likewise, indicated by changes in amino acid profiles (Fig. 5). An observed increase in nearly all amino acids resolved and quantified in the present study (with the notable exception of Ala) is generally consistent with the well-known importance of the liver as the primary site of AA catabolism^51^. More specifically, however, a significant decrease in the ratio of branched chain and aromatic amino acids (BCAA/AAA; Table S1) is highly suggestive of the targeting of hepatocytes. The BCAA/AAA ratio (or “Fischer’s ratio”) is an established biomarker of hepatic damage^52^, and has, in fact, been similarly reported in other, recent studies of hepatotoxins (e.g., aflatoxin-B1^21^; perfluoroalkyl substances^28^) by HRMAS NMR in the zebrafish embryo model.

Although observed alterations of metabolic profiles generally align with targeting of liver, the in vivo visualization of OTA distribution in other organs systems (i.e., kidney, intestine, brain; Fig. 3), and the observed increase of ROS (Fig. 7), in these systems, suggests that hepatocytes are perhaps not the sole cell-types impacted by OTA. Localization of OTA, and observed oxidative stress, in the intestine and kidney is perhaps not surprising given the proximity, and overlapping function, of the liver and these organs as part of the digestive and excretory systems, respectively. And, indeed, nephrocytes and intestinal epithelial cells (alongside hepatocytes) are both demonstrated targets of OTA toxicity^29,30^, and have been shown to be important in the metabolism of OTA^47^.

The observation of increased ROS (and concurrent localization of OTA) to the distal brain region, on the other hand, suggests presumptively independent targeting of the CNS. In fact, both damage to the brain (e.g., hemorrhaging), as well as neurodevelopmental and neurobehavioral effects, have been previously reported for OTA in the zebrafish embryo model^18,24^ and other systems^35^. In the present study, however, *N*-acetylaspartate (NAA), which is found exclusively in neural cells, and is an established biomarker of neural damage^53^, previously linked to neurotoxicity in the zebrafish embryo model^27^, was *not* altered with exposure to OTA (Fig. 5). This observation suggests that, despite localization and apparent elevation of ROS, effects of OTA on neural cells is perhaps not associated with direct cytotoxicity (i.e., neural cell death), or impairment of relevant neural cell functions.

On the contrary, GABA was increased alongside proteinogenic amino acids: the best described function of GABA is its role as a major inhibitory neurotransmitter, as well as a recognized role in neurodevelopment^54^, and altered GABA levels might, at first consideration, align with effects of OTA on neural cells. That said, GABA is, indeed, found in the liver as well, and several studies have pointed to GABA as either a by-product of, or possibly having functional role, in hepatic dysfunction and liver disease^43,55^. And as such, the observed change in GABA levels, in the current study, may simply, in fact, reflect hepatotoxicity of OTA. Alternatively, altered GABA levels may represent an interactive effect between liver and brain: a role of GABA in *hepatoencephalopathy* has been specifically suggested^56^, whereby liver damage leads to the direction of GABA across the blood–brain, and consequently, impaired GABAergic brain function. Further aligned with this, imbalance of the BCAA/AAA ratio (as a biomarker of hepatic damage; discussed above), and altered AAA metabolism specifically, as observed here, has been also suggested to serve a mechanistic role in hepatoencephalopathy^57^, and other neurobehavioral effects^58^. Direct or indirect effects of OTA on neural systems, aside from the observed increase in ROS, however, remain to be seen.

### Effects of OTA on oxidative stress and detoxification pathways

Presumptive targeting of the liver by OTA coincides, in turn, with a previously established role of antioxidant and detoxification pathways^59^ as evidenced, in the present study, by increased ROS (Fig. 7) and reduced GSH (Figs. 5 and 6). Although GSH, for example, is produced by diverse cell types in response to oxidative stress, biosynthesis of the tripeptide is considered perhaps most essential in the liver given its key role phase-II detoxification, i.e., conjugation^44^. Numerous studies have reported effects of OTA on oxidative stress and GSH-based antioxidant and detoxification pathways^44,60^, and several studies have specifically suggested a mechanistic role of Nrf2^13,14,61^. As a transcription factor, Nrf2 binds to antioxidant response elements (ARE) which regulate numerous genes related to antioxidant and detoxification pathways^13^. Reported interactions of OTA and Nrf2 pathways (Fig. 8) include, in this regard, both inhibition of the translocation of the transcription factor to the nucleus^62,63^, and possible direct impairment of binding to ARE^61^. Of the relevant genes regulated by Nrf2/ARE pathways, those involved in both GSH biosynthesis, and phase-II conjugation of xenobiotics including, in particular, glutathione-S-transferase (GST), have been shown to be affected by OTA^13^.

Genes for both steps of GSH biosynthesis in the cytoplasm, namely glutamate-cysteine ligase (GCL) and glutathione synthetase (GS), are regulated by Nrf2^64^. And accordingly, OTA has been recently shown to concomitantly downregulate GCL, and reduce GSH, in hepatocytes via Nrf2/ARE pathways^13^. Blocking of Nrf2 translocation or binding may, furthermore, not only explain reduced GSH, as observed in the present and previous studies, but also perhaps increased levels of the three amino acids, i.e., Glu, Cys and Gly (Fig. 5), from which GSH is biosynthesized. Similarly, both biosynthetic steps are notably ATP-dependent, and the concurrently reduced ATP levels observed for OTA-exposed embryos (Fig. 5) may, therefore, consequently exacerbate the reduction in GSH (and increase in Glu, Cys and Gly precursors). It is specifically proposed based on these observations that depletion of GSH (due to down-regulation via Nrf2, and concurrent ATP depletion) precedes, and consequently leads to, the observed increase in ROS in those cells where OTA is localized, and in turn, consequent effects of oxidative stress including, in particular, disruption of mitochondrial membranes (Fig. 8). The role of OTA-induced GSH depletion in redox homeostasis is, in fact, supported by prior studies: it has been shown in nephrocytes, for example, that while the antioxidant tocopherol reduced ROS levels in OTA-treated cells, GSH levels were unaffected, whereas pre-treatment with *N*-acetyl-l-cysteine (as a glutathione precursor) not only increased GSH, but also reduced both ROS and cytotoxicity^65^.

In addition to the role of GSH in maintaining redox homeostasis, it has been shown that conjugation by, in particular, GST (which is regulated by Nrf2 pathways) may be an important step in the detoxification of OTA, and its phase-I metabolites^44^. It has been previously shown that OTA is metabolized in the liver, kidney and intestines by several phase-I biotransformation pathways including cytochrome P450 (CYP). Notably, phase-I bioactivation can produce metabolites (e.g., lactone-opened and quinone/hydroquinone products) which may, in fact, be more toxic than OTA^66^. Biotransformed metabolites can be subsequently conjugated to GSH (and other hydrophilic moieties, e.g., glucuronic acid) by phase-II detoxification in the liver for removal via intestine and kidney^47^. The concurrent inhibition (via Nrf2) of the expression of GST, as a key enzyme in phase-II conjugation and removal, and the reduced biosynthesis of GSH, therefore, likely represents an important mechanism for potentiating toxicity of OTA in target organs (Fig. 8).

### Role of mitochondria in OTA toxicity

Key consequences of increased cellular ROS include lipid peroxidation and oxidation of membrane proteins which, in turn, lead to disruption of membranes, and loss of membrane function^67^. Accordingly, OTA-induced oxidative stress has, in fact, been previously linked to both peroxidation of lipids and oxidation of proteins within, in particular, mitochondrial membranes^10,61^; and consequent mitochondrial dysfunction is, in turn, one of the earliest events in OTA toxicity. Numerous metabolites altered by OTA exposure in the present study are, likewise, consistent with a role of mitochondrial disruption in the toxicity of OTA (Fig. 8).

Although lipid peroxidation, for example, was not directly measured in the present study, the significant increase in Cho, GPC and m-Ins (Fig. 5), as polar headgroups of membrane phospholipids, is consistent with disruption of membranes as a consequence of oxidative stress. Peroxidation of membrane phospholipids has been consistently linked to hydrolysis, and release of both choline (e.g., GPC) and inositol moieties via both enzymatic (e.g., phospholipase) and non-enzymatic cleavage mechanisms^68^. At least one study, in fact, suggests that phospholipid hydrolysis, rather than preceding lipid peroxidation, is the primary factor in the so-called *mitochondrial permeability transition*^69^. Peroxidation-induced hydrolytic disruption of mitochondrial membranes, in turn, aligns with the consistently observed role of mitochondrial impairment in OTA toxicity which includes disruption of mitochondrial membrane potential (MMP), and consequently reduced ATP, as well as further increased production of ROS, and induction of both mitophagy and mitochondrial biogenesis coupled to disruption of mitochondrial fusion/fission^70,71^. And generally aligned, in the current study, with impaired oxidative phosphorylation (due to disrupted MMP), ATP was significantly decreased by OTA exposure (Fig. 5).

In addition, however, it is proposed (based on altered metabolic profiles) that mitochondrial membrane disruption by OTA simultaneously interferes with uptake, and subsequent metabolism, of several metabolites that are associated with pathways upstream of oxidative phosphorylation (Fig. 8). Reduced uptake, and functioning of these pathways, is likely to result from either general disruption of mitochondrial membrane integrity, or alternatively, oxidation of membrane proteins (i.e., transporters) due to increased cellular ROS. It was, in fact, reported more than four decades ago that OTA inhibits mitochondrial transport systems^72^. And several of the metabolites altered by OTA, in the present study, align with such a mode of action (Fig. 5). The observed decrease in Ala (compared to the significant increase for all other amino acids) is, for example, notable in this regard as it is one of only three amino acids (and the only one which significantly contributes to cellular energetics) not catabolized primarily within mitochondria. Rather, Ala is catabolized by alanine transaminase (ALT) in the cytoplasm of cells (Fig. 8) whereby the transamination of Ala (to Glu) directly generates Pyr for either use in the citric acid cycle, or alternatively, gluconeogenesis^73^. Elevated levels of ALT which is primarily localized to the liver has, in fact, been used as a clinical diagnostic of liver function and dysfunction^73^. Similarly aligned with differential mitochondrial and cytoplasmic metabolism of amino acids, a significant increase was observed for products of both the decarboxylation of Glu to GABA by glutamate decarboxylase (GAD), and amination of Glu to Gln by glutamine synthetase (GS): both enzymatic reactions are, likewise, localized to the cytoplasm, as opposed to other metabolic fates of Glu including conversion to αKG by glutamate dehydrogenase (GDH), and uptake as part of the Mal/Asp shuttle (Fig. 8).

Indeed, several metabolites upstream of oxidative phosphorylation are significantly increased in OTA-exposed embryos suggesting likely impairment of their uptake, and/or subsequent metabolism (Fig. 8). Most conspicuously, in this regard, were significant increases in Mal, Asp, Glu, Glu and αKG (Fig. 5) which are intermediates for interconnected pathways including (1) the malate/aspartate shuttle which transports glycolytic NADH to mitochondria; (2) glutaminolysis whereby Gln taken-up by mitochondria supplies Glu and αKG for both anaplerotic entry to the citric acid cycle, and support of the malate/aspartate shuttle; and (3) the citric acid cycle (Fig. 8). Impairment of all three of these pathways, in turn, would align with observed decrease in both NADH, particularly from the citric acid cycle, and ATP subsequently derived from oxidative phosphorylation. Of the citric acid cycle intermediates resolved by HRMAS NMR, only Mal and αKG were altered; the increase in the latter is particularly revealing, perhaps, as the two irreversible, NADH-generating steps immediately preceding and following this metabolite in the citric acid cycle (i.e., isocitrate dehydrogenase and *α*-ketoglutate dehydrogenase, respectively) represent the two primary regulatory points of the cycle. Further correlated with dysfunction of mitochondrial uptake, both total fatty acids (FA) and Carn were significantly increased. Fatty acids, taken-up by mitochondria, supply precursors (i.e., acetyl CoA) of the citric acid cycle via β-oxidation, whereby carnitine is essential for transport (via carnitine palmitoyltransferases) of fatty acids into mitochondria (Fig. 8). The role of mitochondrial uptake in this observation is highlighted by the observed lack of altered cholesterol: unlike FA, cholesterol is not catabolized by animals (rather removed as bile acids), whereas both are, in contrast, biosynthesized by overlapping pathways (i.e., citrate-derived acetyl CoA) in the cytoplasm.

Concurrent with a putative decrease in mitochondrial uptake and energy metabolism, upstream substrates of carbohydrate metabolism including Glc and associated metabolites were apparently increased (Fig. 5; Supplementary Fig. S1). Although glucose and associated phosphates (i.e., G1P and G6P) could not be unambiguously resolved by 1D HRMAS NMR, the use of 2D COSY confirmed identity of all three metabolites (see Supplementary Fig. S1), and the relative increase of cross-peaks (between treated and control embryos) in these experiments, likewise, consistent with a relative increases in all three metabolites. Increased levels of glucose- associated metabolites would be indicative of either decreased glycolysis, or alternatively, upregulated gluconeogenesis (Fig. 8). Further supporting a role of modulated carbohydrate metabolism, a significant decrease in both Ala and Lac aligns with production of Pyr that could either supply gluconeogenesis, or compensate for reduced glycolytic production. Glucose synthesis from Ala- or Lac-derived Pyr might specifically function in the interplay of energy metabolism between the liver and muscles via Cahill and Cori Cycles which shuttle glycolytic Pyr (from muscle) as either Ala and Lac, respectively, for gluconeogenesis in the liver. That said, however, gluconeogenesis is a highly energy (i.e., ATP) demanding pathway, and considering the presumptive depletion of mitochondrial (i.e., oxidative phosphorylation) generated ATP, it is perhaps unlikely that hepatocytes would favor glucose production and export (to muscle cells) under these conditions. Accordingly, it is more likely that increased Pyr is compensatory for reduced glycolysis. Moreover, recent evidence^71^ points to down-regulation of glycolysis as a result of mitochondrial fusion, and it is posited, as part of our working model (as discussed further below), that OTA may inhibit glycolysis indirectly, and specifically via activation of this mitochondrial repair mechanisms.

The proposed role of mitochondrial repair is based, in part, on a recent study of OTA in intestinal epithelial cells^71^ which demonstrated alongside mitochondrial damage, and concurrent mitophagy and mitochondrial biogenesis altered expression of genes involved in mitochondrial fusion/fission. It was effectively concluded, in this study, that altered mitochondrial fusion/fission, in concert with mitochondrial biogenesis, may serve as a compensatory means to mitigate (by repair and replacement, respectively) mitochondrial damage by OTA. Such a mechanism is, in fact, supported by a prior study in *Drosophila*^74^ which similarly observed an increase in mitochondrial DNA copy number following exposure to OTA. Interestingly, in this regard, it has been recently shown^75^ that Nrf2, as demonstrated target of OTA, plays a key role in the regulation and post-translational modification (i.e., AMP-activated protein kinase) of the genes involved in mitochondrial biogenesis including PPARγ co-activator 1α (PGC1α) and Nrf1, and subsequently, transcription factor A mitochondrial (TFAM); and in turn, was shown that OTA activates the AMPA/PGC1α/TFAM pathway^71^. More relevant to the current study, however, OTA was found in this same study^71^ to lead to altered, and specifically tubular, morphology of mitochondria indicative of fusion. It has, furthermore, been shown that mitochondrial fusion may serve to protect against autophagy and apoptosis, and moreover, support cells during energy deficiency including, in particular, hindered oxidative phosphorylation^76,77^, as observed for OTA. And role of mitochondrial fusion was, likewise, demonstrated in a previous proteomics study of OTA nephrotoxicity which showed downregulation of genes (i.e., OPA1) associated with mitochondrial fusion.

With respect to the present results, it has been recently shown that betaine (which was increased) increases mitochondrial fusion, and alters expression of genes relevant to mitochondrial fusion/fission including increased mitofusin-2 (MFN2) and decreased dynamin-related protein 1 (DRP1), and is, thereby, able to rescue cells from inhibition of oxidative phosphorylation^46^. It is proposed, therefore, that the significant increase in betaine observed, in the present study, may represent a “passive mechanism” to rescue mitochondrial integrity by stimulation of mitochondrial fusion to supplement energy metabolism, in light of mitochondrial dysfunction (and reduced oxidative phosphorylation). At the same time, it was shown in a recent study^78^ that MFN2 interacts with an isoform of pyruvate kinase (PKM2) that serves as key regulator of glycolysis, and serves to coordinate mitochondrial fusion (as a means of mitigating disruptions in oxidative phosphorylation) with the down-regulation of glycolysis. As such, betaine-activated mitochondrial fusion may simultaneously inhibit upstream glycolysis (Fig. 8), consistent with the observed increase in metabolites (i.e., Glc, G6P and G1P) associated with carbohydrate metabolism. A potential compensatory role of betaine in relation to OTA-induced mitochondrial dysfunction would represent not only a previously unknown homeostatic pathway, but also a possibly novel therapeutic target against OTA toxicity.

## Conclusions

In conclusion, our results indicate that OTA induced toxicity mechanism involves compromised detoxification and antioxidant pathways leading to oxidative stress induced mitochondrial dysfunction which are effectively manifested by interconnected cross-talk of energy metabolism and redox imbalance. Based on altered metabolites, a model of OTA toxicity which incorporates the interaction of detoxification/antioxidant and energy metabolism is proposed. As part of this *working model,* it is proposed that under the threat of losing mitochondrial membrane integrity, and functionality in performing oxidative phosphorylation, mitochondrial fusion/fission may provide a compensatory mechanism which may serve as a passive mechanism that may, in turn, be exploited as a possible therapeutic target against OTA toxicity. This working hypothesis, and the proposed model, however, need to be confirmed in future studies. Finally, the affected metabolic processes observed in the present study represent potential *biomarkers* by which possible OTA intoxication and/or exposure can be assessed with respect to human and animal health, and which, likewise, warrant further study.

## Materials and methods

### Chemicals

Ochratoxin A (CAS-number 303-47-9; ≥ 98% (HPLC)) and all other chemicals used in this study were purchased from Sigma-Aldrich (U.S.A.), unless otherwise mentioned.

### Zebrafish embryos

Zebrafish embryos (OBI/WIK line, < 6 hpf) were provided by the UFZ Helmholtz Centre for Environmental Research (Leipzig, Germany). Rearing and breeding of zebrafish (*Danio rerio*), as well as subsequent experimental procedures including exposure, collections and NMR analyses, were performed as described earlier^27,28^, and in accordance with the German animal protection standards approved by the Government of Saxony, Landesdirektion Leipzig, Germany (Aktenzeichen 75-9185.64), and guidelines of the European Union, Directive 2010/63/EU which permits the use of zebrafish embryos up to 120 hpf. All reporting of studies involving use of zebrafish embryos follow the Animals in Research: Reporting In Vivo Experiments (ARRIVE) guidelines^79^.

### Zebrafish embryo toxicity assays

To evaluate embryotoxicity, post-hatch zebrafish embryos (72 hpf) were exposed to concentration of OTA up to 1.0 µM (i.e., 0, 0.25, 0.5, 0.75 and 1.0 µM) in 35-mm diameter polystyrene dishes (*N* = 6 replicates per treatment group, and 10 embryos per replicate). Subsequently, embryos were observed at 24 h, 48 h and 72 h, specifically using a Zeiss CKX41 inverted microscope with phase contrast optics (Olympus, Germany), to measure lethality (i.e., percent survival), and score teratogenic effects (i.e., developmental deformities). Percent survival and teratogenicity was measured, as previously described^17^, as the percent dead or deformed embryos per total number of embryos in each replicate, at each test concentration and each daily observation time point (i.e., 24, 48 and 72 h). Relative (i.e., average) percent mortality and development toxicity at each concentration, and observational time point, were compared by analysis of variance (ANOVA) using OriginPro v. 8 (OriginLab, USA) to determine statistical significance relative to negative (i.e., untreated embryo) controls.

### Visualization of OTA distribution in embryos

The uptake and distribution of OTA in the zebrafish embryos was visualized by laser-scanning confocal microscopy. Embryos, both untreated and treated (with 4.0 µM OTA), were washed with Milli-Q water, and placed on a borosilicate glass coverslip slide in a solution containing embryo medium^80^ with Tricaine anaesthetic (i.e., 1 mg/mL). After 3 min (to assure anaesthesia), images were captured using inverted laser-scanning confocal microscope (Leica DMi8/TL LED, Leica Microsystems CMS GmbH) with a 380-nm laser for excitation, and emission filter set to 460-nm band pass. Images were acquired with a Leica HC PL Apo CS2 (5x/0.15 Dry) objective, and Leica Application Suite X (LAS X) software package version 3.1.5. For quantitative analysis of OTA fluorescence, the images were exported and further analyzed in ImageJ software (ImageJ, USA). Using color deconvolution, the colors were unmixed, and the areas were selected, and subsequently, the number of particles were calculated by the Image-based Tool for Counting Nuclei. Any alterations in brightness and contrast were equally applied to the entire image set. The quantification of OTA fluorescence was performed, and data were exported to Origin Pro v. 8 software for further analysis.

### Quantitation of glutathione in extracts from zebrafish embryos

Glutathione (GSH) levels were measured for extracts from both OTA-treated, and untreated control, zebrafish embryos (to supplement HRMAS NMR measurements). Alongside untreated controls, 72-hpf embryos (*N* = 6 replicates per treatment group**,** and 10 embryos per replicate) were exposed 1.0 µM OTA for 24 h. Embryos (at 96 hpf) were collected and extracted in 5% 5-sulfosalicylic acid. Extracts were centrifugation at 5000 rpm for 5 min, and the supernatant used for GSH quantification using an assay kit (Sigma-Aldrich, U.S.A.), as per the manufacturers' instruction. Briefly, control and OTA-treated (1.0 µM) embryo extracts (10 µl each) were mixed with 150 μL of a working solution, consisting of reaction buffer, diluted enzyme (GSH reductase) and 5,5′-dithiobis-2-nitrobenzoic acid (DTNB), in a 96-well plate. The mixture was incubated for 5 min at room temperature, and subsequently, 50 µl of NADPH solution (160 µg/ml) was added to each well, and incubated again for 20 min. Reduced GSH causes a continuous reduction of DTNB to 5-thio2-nitrobenzoic acid (TNB), and the GSSG formed is recycled by glutathione reductase and NADPH. The yellow product (i.e., TNB) was measured spectrophotometrically at 412 nm using a multiwell-plate fluorimeter (Tecan Infinite 200 Pro, Switzerland).

### Visualization of ROS generation

Generation of ROS was visualized in intact zebrafish embryos following 24 h exposure (at 72 hpf) to 1.0 µM OTA, alongside untreated controls, as described previously^27^. Briefly, 96-hpf embryos were treated in dishes with 1 mM (in 4% DMSO) of the nonfluorescent cell-permeative probe, chloromethyl-2′,7′-dihydrodichlorofluorescein diacetate (CM-H2DCFA; InvitrogenTM LSC6827) to a final concentration of 10 µM, and incubated for 60 min. Embryos were washed 3 times with egg water to remove excess CM-H2DCFA in the medium. Intracellular esterases subsequently cleave the acetate groups of CM-H2DCFA, and the nonfluorescent dye, 2′,7′-dichlorofluorescein (DCF), is formed; when oxidized by intracellular ROS, DCF becomes fluorescent. Embryos were placed on a borosilicate glass coverslip slide in a solution containing embryo medium with Tricaine (1 mg/mL) anaesthetic. Images were captured, after a 3 min delay to ensure a steady-state level of anaesthesia, using inverted laser-scanning confocal microscope (Leica DMi8/TL LED, Leica Microsystems CMS GmbH) with an excitation wavelength of 485 nm, and emission wavelength of 530 nm, using a Leica HC PL Apo CS2 (5x/0.15 Dry) objective and Leica Application Suite X (LAS X) software package, version 3.1.5, to acquire images.

### HRMAS NMR analysis

All HRMAS NMR experiments for metabolic profiling were carried out using a Bruker DMX 600 MHz NMR spectrometer operating with a proton resonance frequency of 600 MHz, which was equipped with a 4 mm HRMAS dual ^1^H/^13^C inverse probe with a magic angle gradient and spinning rate of 6 kHz. Measurements were done at a temperature of 277 K using a Bruker BVT3000 control unit. Acquisition and processing of data were done with Bruker TOPSPIN software (Bruker Biospin GmbH, Germany).

The experiments were performed as adapted from previous studies^27^. Embryos (72 hpf) were treated with 1.0 µM OTA for 24 h by dilution of stock OTA solutions into test medium; control embryos were exposed to equivalent concentration of the solvent vehicle (*i.e.,* ethanol) only. Exposures were performed in polystyrene Petri dishes (100 × 20 mm; Greiner Bio-One) containing 50 mL of test solution with approximately 100 embryos. Additional exposure replicates were prepared in order to generate a sufficient number of embryos (n = 100 per replicate) for quantitative NMR analyses, and account for any loss of embryos due to lethality, since the exposure concentration for OTA was nearly equal to the LC_50_. Accordingly, 6 replicates with 100 embryos per replicate were collected (after 24 h, i.e., 96 hpf) from both controls, and *pooled* OTA-exposures. Embryos were transferred to 4 mm zirconium oxide rotors (Bruker Biospin GmbH, Germany) after they were washed 3-times with MilliQ water to remove residual OTA. Deuterated phosphate buffer (10 µL of 100 mM, pH 7.0) containing 0.1% (*w*/*v*) 3-trimetylsilyl-2,2,3,3-tetradeuteropropionic acid (TSP) was added as a reference to set ^1^H chemical shift at 0 ppm. The rotor was transferred immediately to the NMR spectrometer.

A zgpr pulse sequence (from Bruker’s standard pulse program library) with water suppression was used for one-dimensional ^1^H HR-MAS NMR spectra. Each one-dimensional spectrum was acquired by applying a spectral width of around 12,000 Hz, domain data points of 8 k, a number of averages of 128 with 0 dummy scans, a constant receiver gain of 256, an acquisition time of 2 s, and a relaxation delay of 0.17 s. In order to avoid the effects of short *T*_*2*_ components due to the presence of lipids in intact embryo samples, the relaxation delay was set to a small value. All spectra were processed by an exponential window function corresponding to a line broadening of 1 Hz and zero-filled before Fourier transformation. NMR spectra were manually phased and automatically baseline corrected using TOPSPIN 4.0.6 (Bruker Biospin GmbH, Germany). The total analysis time, including sample preparation, optimization of NMR parameters, and data acquisition of ^1^H-HRMAS NMR spectroscopy for each sample was approximately 20 min.

### HRMAS NMR data analysis

The spectra were, analyzed by using TOPSPIN 3.1. For the quantification of metabolites in the HR MAS NMR spectrum, Chenomx NMR Suite 8.2 (Chenomx Inc., Canada) was used which provides qualitative and quantitative analysis of NMR spectra by fitting spectral signatures from the Human Metabolome Database (HMDB). Using the *Profiler* module of Chenomax, a Lorentzian peak shape model of each metabolite is generated from the HMBD information, and superimposed upon the actual spectrum. The area of the peaks from each metabolite is directly related to its abundance. The linear combination of all metabolites gave rise to the total spectral fit, which can be evaluated with a summation line (see Supplementary Fig. S2). The concentrations of metabolites were calculated as a ratio relative to tCr as an internal reference as described previously^21^. NMR quantification was statistically analyzed by means of one-way ANOVA with a Tukey post-hoc correction for multiple comparisons. A *P*-value of < 0.05 was considered significant. To check the false discovery rate, the p-values were corrected for multiple testing, and q-values were obtained using the Benjamini–Hochberg method^81^. Levene’s test was performed for homogeneity of variance analysis, and indicated that the population variations were not significantly different.

For multivariate analysis, SIMCA software package (Version 14.0, Umetrics, Sweden) was used. From the one-dimensional NMR spectra of control and OTA-treated embryos, the bucket tables were generated using MestReNova v.12.0.4 (Mestrelab research S.L., Spain). The one-dimensional spectra were normalized to the total intensity and subdivided into buckets of 0.04 ppm, after removing the region between 4.20 and 6.00 ppm to exclude the larger water signal. The data were mean-centered and scaled using the Pareto method in the SIMCA software package. Supervised partial least square-discriminant analysis (PLS-DA) was then performed on the data using the SIMCA software as described earlier^27^. Five components were calculated for the model. The PLS-DA model was further validated using the permutation test (using 150 permetuation) with SIMCA (see Supplementary Fig. S3).

## Supplementary Information

Supplementary Information.

## Data Availability

All data generated or analysed during this study are included in this published article (and its Supplementary Information files). Additional raw data files can be available from the corresponding author on request.
