# Supplementary material for: An integrated systems-level model of ochratoxin A toxicity in the zebrafish (Danio rerio) embryo based on NMR metabolic profiling
Source: Sci Rep. 2022 Apr 15;12:6341. doi: 10.1038/s41598-022-09726-4 (PMC9012740; doi:10.1038/s41598-022-09726-4)
Supplement: Supplementary file 1 — Supplementary Information. [file 41598_2022_9726_MOESM1_ESM.docx]

**Supplementary Materials**

**Table S1.** Relative change in branched chain (BCAA) and aromatic (AAA) amino acids measured by HRMAS NMR in zebrafish embryos exposed to Ochratoxin A, versus controls. Embryos exposed to 1µM OTA; negative controls exposed to solvent vehicle. Given are mean concentrations (mm, normalized to total creatine), and standard deviation (SD), as well percent change (%Change) in metabolite (relative to control). Significant difference indicated by calculated p-values from ANOVA.

|  |  | **Concentration (mM)^b^** | | | | | | | | | | |  |  |
| --- | --- | --- | --- | --- | --- | --- | --- | --- | --- | --- | --- | --- | --- | --- |
|  | **Metabolite^a^** | **Control ± SD** | | | |  | **OTA ± SD** | | | | | **%Change** | | ***p*-Value** |
|  |  |  |  | |  |  |  | |  | |  | |  |  |
| ***AAA*** | | | | | |  |  |  | |  | | |  |  |
|  | Phe | 0.27 | ± | | 0.02 |  | 0.41 | | ± | | 0.05 | | 48.1% | <0.05 |
|  | Tyr | 0.25 | ± | | 0.01 |  | 0.59 | | ± | | 0.09 | | 136% | <0.05 |
|  | Trp | 0.43 | ± | | 0.04 |  | 1.04 | | ± | | 0.17 | | 141.8% | <0.001 |
|  |  |  |  | |  |  |  | |  | |  | |  |  |
| ***BCAA*** | |  | |  | |  |  |  | |  | | |  |  |
|  | Leu | 1.45 | ± | | 0.06 |  | 2.06 | | ± | | 0.09 | | 42.0% | <0.05 |
|  | Ile | 0.72 | ± | | 0.05 |  | 1.70 | | ± | | 0.21 | | 136.1% | <0.05 |
|  | Val | 0.54 | ± | | 0.05 |  | 0.86 | | ± | | 0.05 | | 59.1% | <0.05 |
|  |  |  |  | |  |  |  | |  | |  | |  |  |
| ***BCAA/AAA*** | | 2.85 | ± | | 0.05 |  | 2.26 | | ± | | 0.17 | | 79.3% | <0.05 |
|  | |  |  | |  |  |  | |  | |  | |  |  |

**
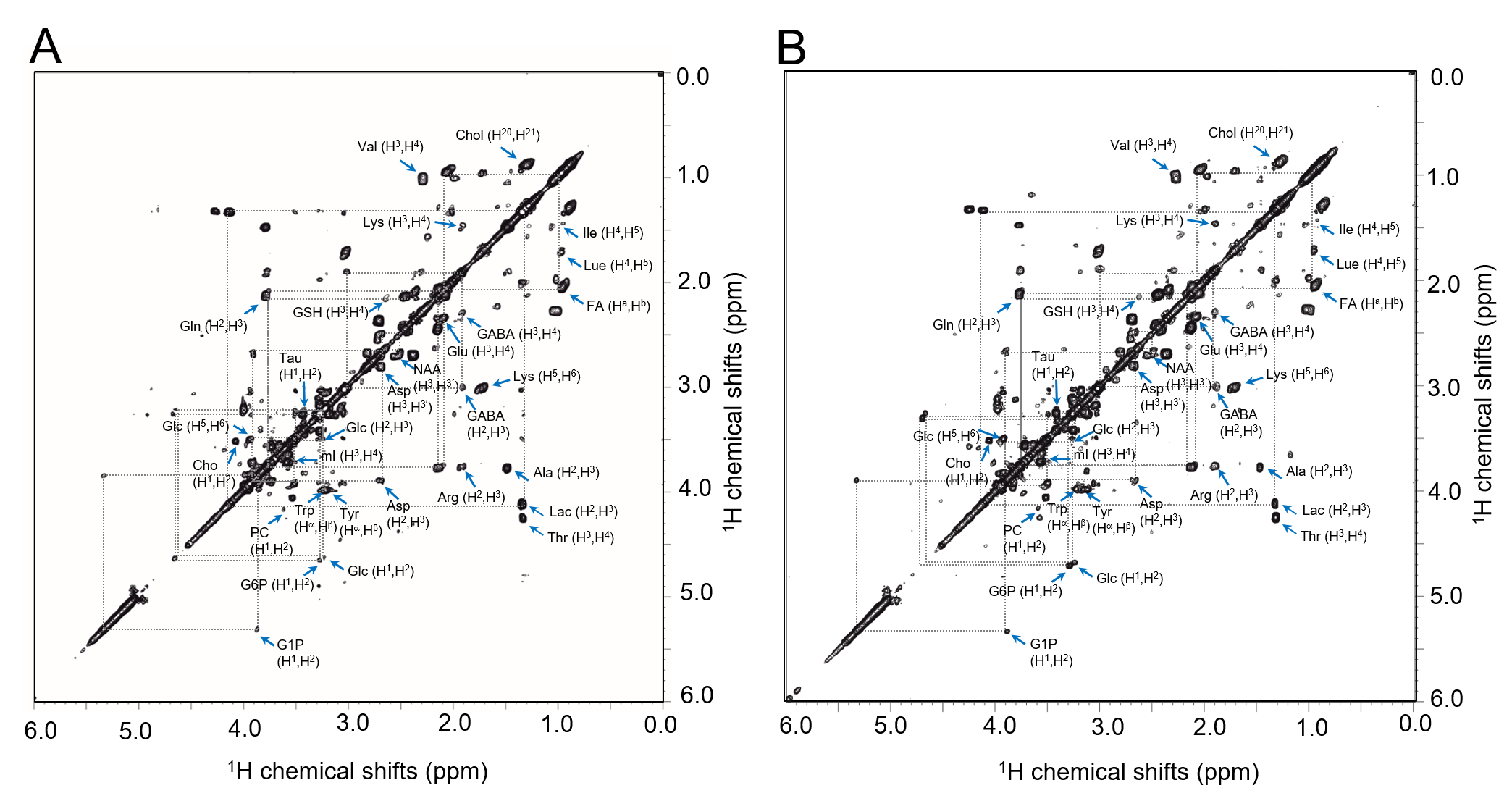
**

**Supplementary Fig. S1.** Comparison of ^1^H-^1^H COSY spectra of control (A) and 1µM OTA treated (B) embryo in the range of 0 to 6 ppm. Measurement of ^1^H-^1^H COSY spectra and assignment of the cross peaks is made according to ref [27]. The parameters used for COSY were 2048 data points collected in the t2 domain over the spectral width of 9k, 512 t1 increments were collected with 16 transients, relaxation delay 2 sec, acquisition time 114 msec, and pre-saturated water resonance during relaxation delay. The resulting data were zero filled with 512 data points and were weighted with the squared sine bell window functions in both dimensions prior to Fourier Transformation. Application of gradient pulses along with tradition ^1^H-^1^H COSY sequence provides highly resolved spectrum. Changes in cross peak areas of many metabolites in OTA treated embryos, as compared to control, align well with the results obtained from quantitative estimation of metabolites from 1D spectra based on Chenomx as shown in Fig. 5. For example, a decline in cross peak area of lactate (Lac), alanine (Ala) and glutathione (GSH), and increase in the cross peak area of several metabolites including glucose (Glc), glucose-6-phosphate (G6P) and glucose-1-phosphate (G1P) in OTA treated embryos (as compared to control) is clearly visible. These results agree well with the quantitative estimation of these metabolites shown in Fig. 5.

**
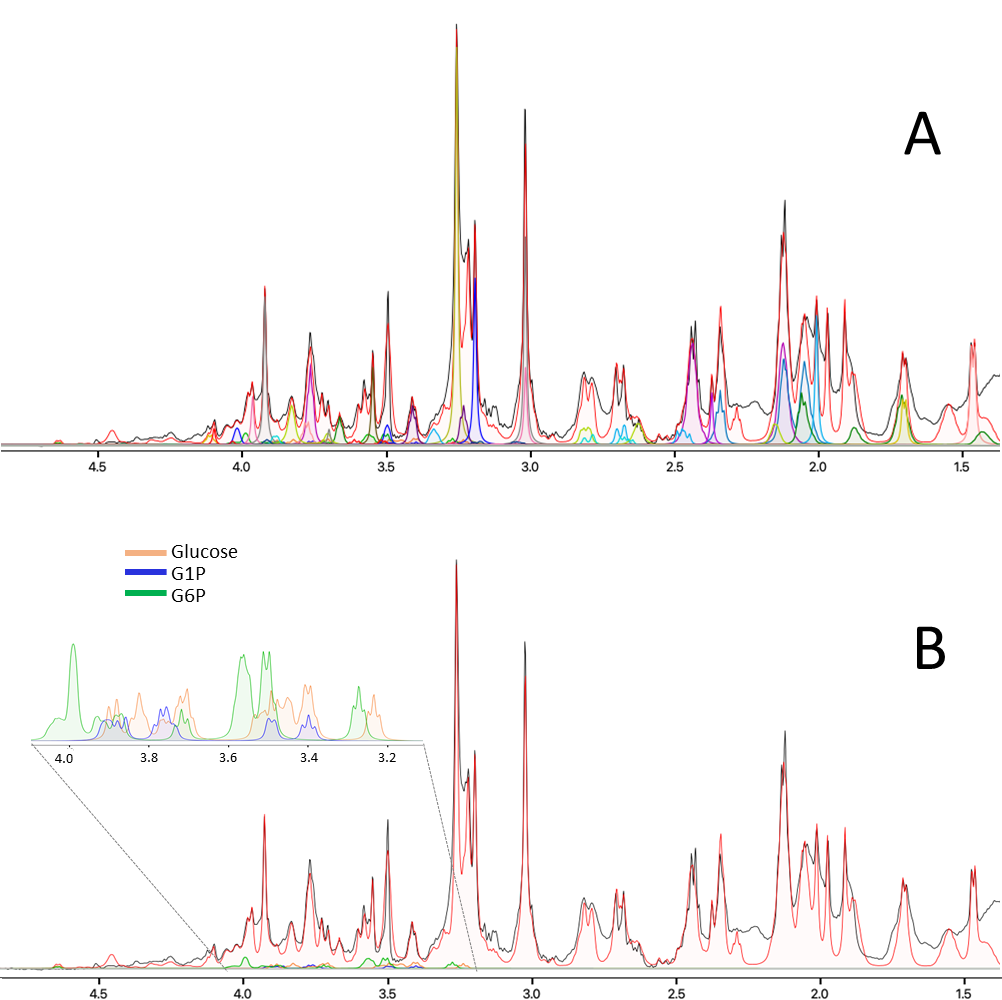
**

**Supplementary Fig. S2.** **(A)** Example of metabolite line fitting simulations of ¹H HRMAS NMR spectra from Zebrafish embryos using Chenomx NMR Suite 8.2 (Chenomx Inc., Edmonton, Alberta, Canada). **(B)** The spectrum showing example of the part of the fittig simulation of glucose, glucose-1-phosphate (G1P) and glucose-6-phosphate (G6P) between the region of 3.1 and 4.1 ppm.

####
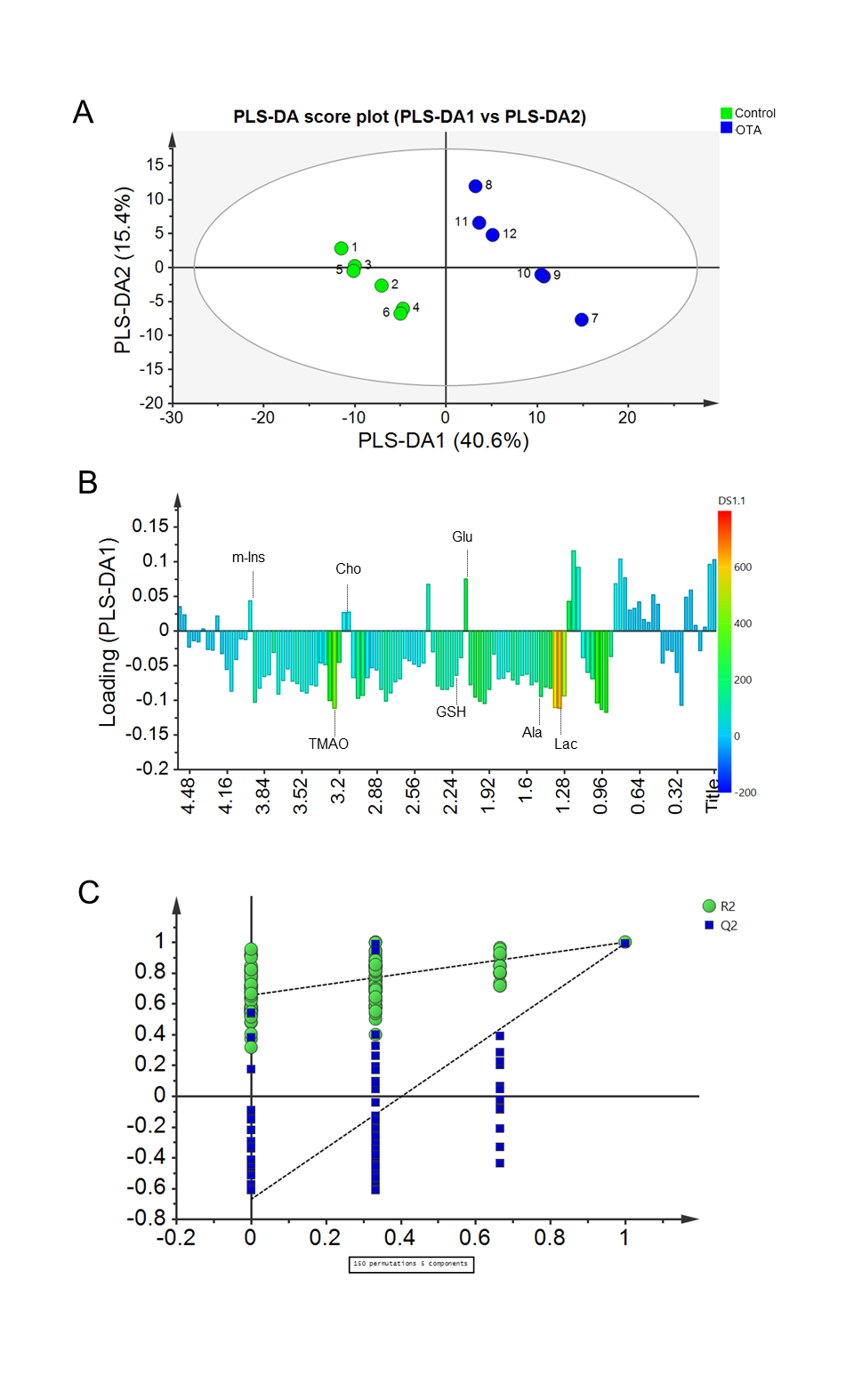


**Supplementary Fig. S3.** Multivariate analysis of the HR-MAS NMR spectra (n=6 per group) using partial least square-discriminant analysis (PLS-DA) modelling (R2 = 0.999, Q2 = 0.979). (A) Scores plots (PLS-DA1 vs PLS-DA2). The score plot explains 56% of total variance of control clustering in the negative PLSDA1 scores, and OTA in the positive PLSDA1 scores. (B) Loading plots of PLS-DA1 for all buckets containing assigned peaks. (C) Permutation plot to test PLS-DA model (number of components 5; permutation 150). A permutation test was used to check PLS-DA model validity. The X variables were kept intact, while the Y variables were permuted and then the original variables were compared to the permuted variables. The plot in (c) strongly indicates that the original model is valid. The criteria for validity are that: all blue Q2-values to the left are lower than the original points to the right; & the blue regression line of the Q2-points intersects the vertical axis (on the left) at (0, -0.671). This indicates the good quality of the model.
